# Emerging Infectious Diseases in Pregnant Women in a Non-Endemic Area: Almost One Out of Four Is at Risk

**DOI:** 10.3390/pathogens10010056

**Published:** 2021-01-10

**Authors:** Giulia Modi, Beatrice Borchi, Susanna Giaché, Irene Campolmi, Michele Trotta, Mariarosaria Di Tommaso, Noemi Strambi, Alessandro Bartoloni, Lorenzo Zammarchi

**Affiliations:** 1 Department of Experimental and Clinical Medicine, University of Florence, Largo Brambilla 3, 50134 Florence, Italy; giuliamodi7794@gmail.com (G.M.); alessandro.bartoloni@unifi.it (A.B.); 2 Referral Centre for Infectious Diseases in Pregnancy of Tuscany, Largo Brambilla 3, 50134 Florence, Italy; borchib@aou-careggi.toscana.it (B.B.); susanna.giac@hotmail.it (S.G.); campolmii@aou-careggi.toscana.it (I.C.); trottam@aou-careggi.toscana.it (M.T.); 3 Infectious and Tropical Diseases Unit, Careggi University and Hospital, Largo Brambilla 3, 50134 Florence, Italy; 4 Department of Health Sciences, Obstetrics and Gynaecology Branch, University of Florence, Largo Brambilla 3, 50134 Florence, Italy; mariarosaria.ditommaso@unifi.it (M.D.T.); noemi.strambi@gmail.com (N.S.); 5 Referral Centre for Tropical Diseases of Tuscany, Largo Brambilla 3, 50134 Florence, Italy

**Keywords:** Chagas disease, HTLV, malaria, schistosomiasis, Zika, pregnancy

## Abstract

We report the results of a targeted testing strategy for five emerging infectious diseases (Chagas disease, human T-lymphotropic virus 1 infection, malaria, schistosomiasis, and Zika virus infection) in pregnant women accessing an Italian referral centre for infectious diseases in pregnancy for unrelated reasons. The strategy is based on a quick five-question questionnaire which allows the identification of pregnant women at risk who should be tested for a specific disease. One hundred and three (24%) out of 429 pregnant women evaluated in a 20 month period were at risk for at least one emerging infectious disease. Three (2.9%, all from sub-Saharan Africa) out of 103 at-risk women resulted in being affected (one case of *Plasmodium falciparum* malaria, two cases of schistosomiasis) and were appropriately managed. Prevalence of emerging infectious disease was particularly high in pregnant women from Africa (three out of 25 pregnant women tested, 12%). The proposed strategy could be used by health care professionals managing pregnant women in non-endemic setting, to identify those at risk for one of the five infection which could benefit for a targeted test and treatment.

## 1. Introduction

Chagas disease (CD), human T-lymphotropic virus 1 (HTLV-1) infection, malaria, schistosomiasis, and Zika virus (ZIKV) infection are five emerging infectious diseases having a relevant impact on pregnancy outcome, despite them often being asymptomatic or paucisymptomatic in pregnant women [1,2,3,4,5,6]. Reliable diagnostic and treatment options to manage these infections during pregnancy or in the immediate post-partum period are available [7,8,9,10,11,12,13,14]. The increase in international travels as well as migrations is considerably modifying the epidemiology of these tropical and subtropical infections, and nowadays they may be easily observed in temperate high-income countries [15,16,17]. For example, in Italy around 6 million of migrants are currently present (8.9% of resident population), most of them coming from Europe (mostly from Romania, Albania, Ukraine, and Moldova), Africa (mostly from Morocco, Egypt, Nigeria and Senegal), Asia (mostly from China, Philippines, India, and Bangladesh), and Latin America (mostly from Peru, Ecuador, Brazil, and Dominican Republic) [18,19,20].

Main clinical characteristics of CD, HTLV-1 infection, malaria, schistosomiasis, and ZIKV infection and recommended management strategies in pregnant women and their neonates are summarized in Table 1.

Testing for CD, HTLV-1 infection, malaria, schistosomiasis, and ZIKV infection during pregnancy is variously recommended by different guidelines and in different settings, but not systematically implemented in the majority of temperate countries [7,8,9,10,11,12,13,14].

According to these guidelines we recently implemented a questionnaire based targeted testing approach for CD, HTLV-1, malaria, schistosomiasis, and ZIKV for pregnant women attending the Tuscany Referral Centre for Infectious Diseases in Pregnancy (TRCIDP), Azienda Ospedaliera Universitaria of Careggi, Florence, Italy in collaboration with the Tuscany Reference Centre for Tropical Diseases, Azienda Ospedaliera Universitaria of Careggi, Florence, Italy. The TRCIDP is located in a tertiary hospital and every year receives between 200 and 300 women for evaluation of possible or confirmed infections in pregnancy.

According to the Italian national recommendation, in our setting the standard infectious disease screening during pregnancy includes serology for Rubella, *Toxoplasma gondii*, HCV, HBV, HIV, syphilis, and vaginal swab for group B streptococcus. Moreover, screening for CD in continental Latin American women and women whose mother was born in continental Latin America is also formally recommended by the Tuscany region, but still not fully implemented [33]. Most of referrals to TRCIDP are due to one of these suspected or confirmed infections.

Here we report a descriptive analysis of data collected after the first 20 months (March 2019–November 2020) of the questionnaire based targeted testing approach for CD, HTLV-1, malaria, schistosomiasis, and ZIKV for pregnant women, implemented at the TRCIDP.

## 2. Materials and Methods

**Figure 1:**
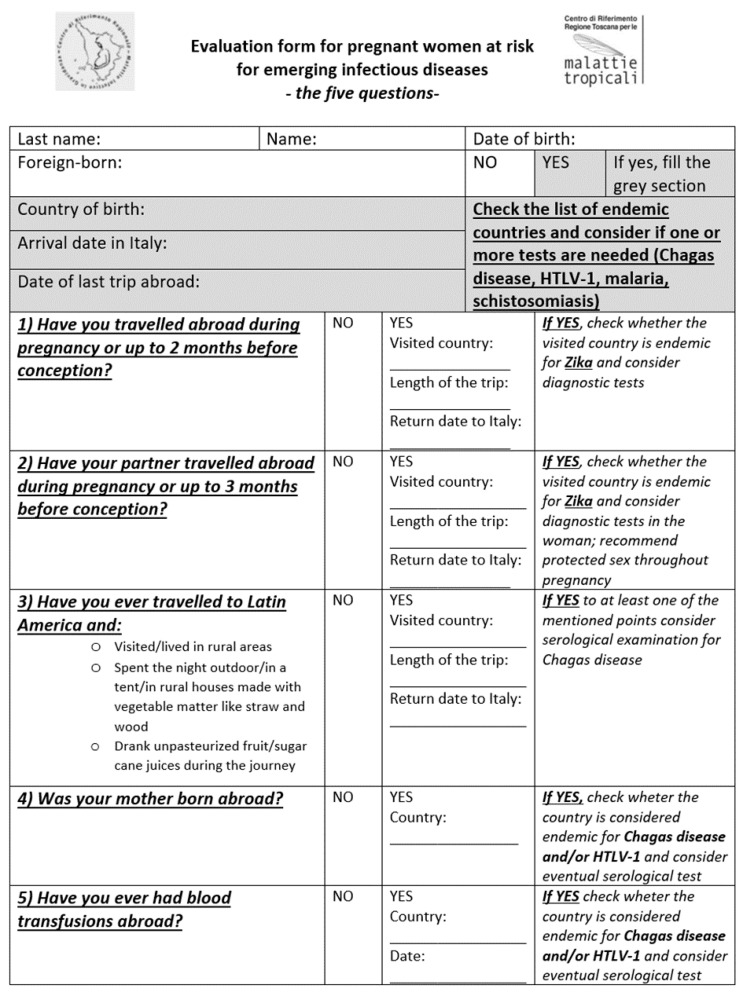
Questionnaire used to evaluate the presence of potential risk factors for emerging infectious diseases in pregnant women attending the centre.

Since March 2019, pregnant women accessing TRCIDP for any reason are asked to answer a short questionnaire evaluating potential risk factors for CD, HTLV-1 infection, malaria, schistosomiasis, and ZIKV infection (“Evaluation form for pregnant women at risk for emerging infectious diseases”, Figure 1) in addition to the usual evaluation for the main reason of the visit. If the woman does not present any of the risk factors listed in the questionnaire, no further investigations will be performed other than those provided for in the basic protocol. The questionnaire is administered directly by the managing physician in Italian or English or with help of a cultural mediator if needed. Filling the questionnaire and consulting the list of endemic countries takes from 40 s to about 2 min.

If one or more potential risk factors are identified by using the questionnaire, a list of endemic countries for the five diseases (Supplementary Table S1, drawn up in accordance with the epidemiological indications of the main health authorities [22,24,34,35]) is consulted by the physician to confirm that the women is at risk for one or more infections. Only in this case, a specific diagnostic test is performed.

The first section of the questionnaire is focused on personal epidemiological data, mostly the country of birth. In case of foreign-born patient, the grey section is also filled.

The second section consists of five questions inquiring more specific risk factors. Questions number 1 and number 2 aim to investigate the risk of vector and/or sexual exposure to ZIKV infection, both in Italian travelers and migrant women. In case of an affirmative answer to one or both questions, the countries visited, the duration of the trip and the return date to Italy will be recorded. Question number 3 is notably addressed to Italian travelers and those who came from non-endemic countries for CD. The three listed risk factors (a, b, and c) are related to vectorial and oral transmission of *Trypanosoma cruzi*. The fourth question about the country of origin of the patient’s mother is justified by the evidence that CD and HTLV-1 infection may be transgenerationally-transmitted. These two infections could also be transfusion-transmitted in high endemic countries where screening is not performed, hence the interest in the last question. Concerning malaria, only pregnant women born in sub-Saharan Africa (SSA) who have arrived within the previous five years or who have visited their country of origin within the last five years are considered to be at risk. Regarding schistosomiasis, only pregnant women born in high to moderate endemic areas according to the WHO such as SSA countries, Brazil, Philippines, and Yemen are considered at risk.

In women resulting “at risk” for one or more infections, a specific diagnostic test is performed followed by disease specific intervention in case of positivity. Table 2 summarizes the specific test performed and the planned management in pregnant women and their newborns in case of positive results.

## 3. Results

A total of 429 pregnant women coming from 46 different countries were evaluated in the study period. Most of patients were born in Italy (*n* = 284, 66%), the others were born in other European countries (*n* = 69, 16%), North America (*n* = 2, <1%), Latin America or the Caribbean (*n* = 29, 7%), Africa (*n* = 25, 6%), and Asia (*n* = 20, 5%). The most frequent foreign countries of birth were Albania (*n* = 26, 6%), Romania (*n* = 20, 5%) Peru (*n* = 14, 3%), and China (*n* = 9, 2%). The median age was 34 years old (range 17–46). The type and the frequency of potential risk factors for emerging infectious diseases according to the questionnaire answers (Figure 1) are reported in Table 3. One hundred and eighty four (43%) women presented at least one potential risk factor; however, after consulting the list of endemic countries (Supplementary Table S1), some of them were not confirmed to be at risk (for example a women born in Albania was “foreign born”, so potentially at risk; however, Albania is not considered at risk for any of the five emerging infectious diseases).

Overall, 103 pregnant women (24%; 95% CI 20–28) were confirmed to be at risk for at least one of the five emerging infectious diseases and were subsequently tested. The frequency of risk factors for CD, HTLV-1 infection, malaria, schistosomiasis, and ZIKV infection was 10% (95% CI 7.3–13.1; 44 out of 429), 13% (95% CI 9.6–16; 55 out of 429), 3% (95% CI 1.1–4.1; 11 out of 429), 5% (95% CI 3–7.2; 22 out of 429), and 8% (95% CI 5.2–10.2; 33 out of 429), respectively. The frequency of risk factors according to the geographical area of origin is reported in Supplementary Table S2. Among the 103 patients who underwent one or more targeted diagnostic tests, three (2.9%), all from Africa, resulted affected by an emerging infectious disease (one case of *Plasmodium falciparum* malaria and two of schistosomiasis). In detail, positive results were found in one out of 11 (9%) women tested for malaria and two out of 22 (9%) women tested for schistosomiasis. For both infections the number needed to screen was 11. African pregnant women were those who tested positive for at least one disease (three out of 25, 12%).

The patient tested positive for malaria was a 25-year-old woman who had arrived in Italy four years earlier from Nigeria and who had also travelled to Benin three months earlier for two weeks. She accessed the TRCIDP at 25 weeks of gestation for positive Rubella IgM antibodies which was subsequently attributed to a false positive. She was in her second pregnancy and had no symptoms or signs of malaria, in particular no evidence of splenomegaly. As for laboratory exams she had anemia (hemoglobin 8.4 g/dL) but no thrombocytopenia. Nevertheless, loop mediated isothermal amplification (LAMP) was positive and thin blood slide was positive for *P. falciparum* with a parasitemia of 0.1%. She received a 3 days treatment with artemether lumefantrine and fully recovered. The patient tested positive for schistosomiasis was a 31 years old Ethiopian woman, resident in Italy for 5 years. She accessed the TRCIDP for doubtful serology for *T. gondii* (which was subsequently attributed to a false positive result) at 35 weeks of gestation, without any symptoms or signs of other infections. Serological test for schistosomiasis turned out positive, as well as the urine circulating cathodic antigen (CCA Ag) test. Hemoglobin level was slightly reduced (10.7 g/dL) and she had no eosinophilia. Due to the late gestational age and the absence of biological and ultrasound signs of advanced schistosomiasis, she was not treated during pregnancy but shortly after delivery with one dose of praziquantel. The second patient tested positive for schistosomiasis was a 37-year-old woman, in her third pregnancy, who had arrived in Italy in 1999 from Cote d’Ivoire and on follow-up for HBV infection. She had last travelled to Africa four years previously. She accessed the TRCIDP at 37 weeks of gestation to define the management of HBV infection. A serological test for schistosomiasis was positive and laboratory tests showed slight anemia (hemoglobin 11.5 g/dL) and no eosinophilia. In this case, given the advanced gestational age, schistosomiasis was treated after delivery.

## 4. Discussion

The results of this study shows that among the 429 pregnant women evaluated through a five-question questionnaire, almost one out of four presented risk factors for one or more emerging infectious diseases, reaching almost two out of three African women and the totality of continental Latin American women.

The small sample size does not allow an accurate estimate of the prevalence of the single infection in pregnant women at risk, but our preliminary results suggest that emerging infectious diseases should not be overlooked in non-endemic setting.

Notably, the prevalence of emerging infectious diseases seems particularly high in pregnant women from Africa. As a matter of fact, among the minimal sample of 25 African women tested, three (12%) turned out positive for one infection.

High seroprevalence (18.4%) for *Schistosoma* spp infection is expected in migrants from endemic countries, for this reason it is not surprising that the most frequently diagnosed disease was schistosomiasis [36]. Two (9%) out of 22 women tested (16 from SSA and 6 from Brazil) had positive serology and one woman presented CCA Ag positive in urine suggesting active disease. Serological screening for schistosomiasis of migrants coming from endemic areas and treatment of sero-positive subjects is recommended by the majority of international guidelines [10,28,29]. Pregnant women should not be excluded from this screening since the infection may have deleterious effect on maternal, fetal and neonatal outcomes and the World Health Organization (WHO) has recently authorized the administration of praziquantel in pregnancy [27,37].

One (9%) of the 11 SSA pregnant women exposed in their area of origin during the previous 5 year tested positive for malaria using LAMP for *Plasmodium* spp. A prevalence of sub-microscopic malaria ranging between 3 and 9% in asymptomatic SSA migrants recently arrived in Spain and Canada has been reported [38,39]. Our patient had a mild anemia and a low *P. falciparum* parasitemia, without fever or other symptoms. Severe anemia, often without symptoms, is a common feature of malaria in pregnancy (especially in primigravidae) in the population living in endemic areas with stable transmission which develop some degree of immunity after reiterate exposure [40]. This “semi-immunity” is also responsible for the prolonged incubation period in migrant subjects [41]. Episodes of symptomatic *P. falciparum* malaria in migrant pregnant women are reported up to 4 years after their last exposure in endemic areas, further justifying the screening for this disease during pregnancy [42]. The “Domestic Refugee Health Guidelines” by the Centers for Disease Control and Prevention recommend malaria PCR screening in all asymptomatic pregnant refugees originating from SSA who have not been treated before arriving in the US [12].

No cases of CD, HTLV-1 infection, and ZIKV infection were diagnosed in our population, despite 44 (10%), 55 (13%), and 33 (8%) of women being at risk for the three conditions, respectively. The low yield of testing is probably attributable to the small sample and to the relatively low prevalence of these diseases.

The estimated prevalence of CD in continental Latin American pregnant women delivering in Italy is about 1.7% [43]. Concerning HTLV-1 infection, the expected prevalence in pregnant women coming from endemic countries but residing in Europe may be up to 1.64% in women from the Caribbean, 0.96% in women from SSA, 0.43% in women from Japan and 0.2% in women from South America [44]. Concerning ZIKV infection, 2.9% (18 out of 621) of symptomatic and 0.3% (7 out of 2425) of asymptomatic pregnant women tested in the US after travelling in an affected area resulted infected [45]. However, the study was referred to 2016 when ZIKV pandemic was on the rise. The risk of acquiring ZIKV infection is currently lower due to the more limited circulation of the virus.

Despite the relatively low prevalence of these three infections in pregnant women, several recommendations are in favor of screening during pregnancy due to the potentially related poor outcome, the availability of effective intervention and, for some of these diseases, the favorable cost-effectiveness profile [2,9,14,46].

Our study has several limitations, first of all the small sample size and the fact that pregnant women attending the TRCIDP may not be representative of the general pregnant population in Italy or in other non-endemic settings, although none of the patients were specifically sent for suspicion of an emerging infectious disease. Even thus the questionnaire was easily administered by physicians of the TRCIDP the feasibility was not formally investigated. Moreover, some risk factors (like history of use of intravenous drugs or having multiple sex partners, risk factors for HTLV-1 infection) were not inquired.

## 5. Conclusions

Our results preliminarily suggest that a questionnaire-based testing strategy for emerging infectious diseases in pregnancy could allow the quick identification of pregnant women at risk in which testing for these infections is recommended. Overall prevalence of risk factors for at least one of the selected diseases was considerably high in our study, with almost one out of four women being at risk. Moreover, three infected women (one with *P. falciparum* malaria and two with schistosomiasis) who would otherwise go unnoticed, were promptly detected and treated. Additional data are needed to ascertain whether this type of questionnaire could be useful for health care professionals managing pregnant women in non-endemic setting, in order to easily identify those at risk for one of the five infections which could benefit from a targeted test.

## Figures and Tables

**Table 1:** Main clinical and epidemiological characteristics of Chagas disease, human T-lymphotropic virus 1 infection, malaria, schistosomiasis, and Zika virus infection in pregnant women and their neonates and recommended management strategies.

Emerging Infectious Diseases	Main Epidemiological Features	Rate of Mother to Child Transmission	Main Clinical Consequences on Maternal and Neonatal Health	Recommended Management in Pregnant Women and Their Newborns
Chagas disease	Endemic in continental Latin America.	≈4% in non-endemic setting [21].	Chagasic cardiopathy and intestinal megasyndromes in ≈30% of infected subjects.	Serological screening in pregnant women at risk. Testing and follow-up of their children. Antiparasitic treatment of the infected children within 1 year of life. Antiparasitic treatment of infected women by the end of breastfeeding [2].
Human T-lymphotropic virus 1 infection	Higher prevalence in several sub-Saharan African and Latin American countries, Japan, Iran, Romania [22].	15–20% (mainly through breastfeeding) [9].	Adult T-cell leukemia/lymphoma or HTLV-1-associated myelopathy/tropical spastic paraparesis in <10% of infected subjects [23].	Serological screening in pregnant women at risk. Short term breast feeding or breastfeeding avoidance in HTLV-1 infected women [9].
Malaria	Endemic in several tropical and sub-tropical countries. 90% of cases occur in Africa [24].	Congenital malaria occurs in <1–6% of cases [25].	Increased incidence of maternal anemia, LBW, IUGR, stillbirth, miscarriage, congenital malaria [26].	Testing with polymerase chain reaction for malaria all pregnant refugees originating from sub-Saharan Africa not receiving pre-departure therapy [12]. Treatment of affected women.
Schistosomiasis	Endemic in several tropical and sub-tropical countries. 85% of cases occur in Africa.	Congenital transmission is only anecdotally reported.	Increased incidence, of maternal anemia, LBW and prematurity [27].	Serological screening of migrants from endemic countries and antiparasitic treatment of those seropositive [10,28,29].
Zika virus infection	Causes outbreak in several tropical and subtropical countries.	Up to 65% [30].	Reported risk of CZS (characterized by microcephaly and other clinical features) is highly variable (from 1 to 8%) in neonates from pregnant women with confirmed infection [31,32]	Testing of all symptomatic and asymptomatic pregnant women returning from an at risk area or sexually exposed to a partner recently returned from an at risk area [14].

Footnotes: LBW = Low birth weight; IUGR = intrauterine growth restriction; CZS: congenital Zika syndrome.

**Table 2:** Diagnostic test used in pregnant women at risk for selected emerging infectious diseases at the Tuscany Referral Centre for Infectious Diseases in Pregnancy, Azienda Ospedaliera Universitaria of Careggi, Florence, Italy, and planned management.

Disease	Diagnostic Test Performed in Pregnant Women at Risk	Management of Pregnant Woman in Case of Positive Results	Suggested Management of the Newborn in Case of Positive Results
Chagas disease	*Chemiluminescent Microparticle Immunoassay* (CMIA), Architect Chagas^®^, Abbott Laboratories, Wiesbaden, Germany.	Confirmation with a second different serological test. Clinical and instrumental evaluation (electrocardiogram and echocardiogram) of the women to assess presence of cardiac or gastrointestinal involvement. Treatment with benznidazole or nifurtimox of the women after breastfeeding.	Clinical and laboratory evaluation at birth. Referral to a pediatric infectious diseases centre for clinical and laboratory follow-up.
Human T-lymphotropic virus 1 infection	CMIA, Architect rHTLV-I/II, Abbott Laboratories, Wiesbaden, Germany.	Confirmation with Western Blot (WB) and proviral load determination.Infected women should avoid breastfeeding, preferring formula milk feeding, frozen-thawed breast milk or short-term breastfeeding (≤3 months).	Referral to a pediatric infectious diseases centre for clinical and laboratory follow-up.
Malaria	*Loop mediated isothermal amplification* (LAMP), Alethia™ Malaria, Launch Diagnostics, Longfield, United Kingdom.	Microscopy is necessary for confirmation and definition of parasitemia; PCR real time for the determination of the species.Infected women will receive antimalarial treatment according to severity, *Plasmodium* species and gestational age.	Clinical and laboratory evaluation at birth and follow-up.
Schistosomiasis	*Western blot* (WB), LD-BIO Diagnostics, Lyon, France.	Parasitological exams of urine (three samples) and stools (three samples) and determination of CCA (*circulating cathodic antigen*) are needed to confirm active parasitosis.Treatment with praziquantel can be programmed during the second/third trimester of pregnancy or after childbirth.	Clinical evaluation and follow-up.
Zika virus infection	Serological test (ELISA ZIKV IgG-IgM, Euroimmun AG, Luebeck, Germany) and a molecular test for virus identification in serum and urine (Zika Virus Real Time RT-PCR Kit, Liferiver/Shanghai ZJ Biotech Co. China).	Serial fetal ultrasounds; Possibility of amniocentesis; possibility of VTP according to current legislation.	Clinical and laboratory evaluation at birth. Referral to a pediatric infectious diseases centre for clinical and laboratory follow-up.

VTP: Voluntary Termination of Pregnancy; CZS = Congenital Zika Syndrome.

**Table 3:** Type and frequency of potential risk factors for emerging infectious diseases according to the questionnaire results.

Type of Potential Risk Factor	Frequency of Potential Risk Factor
Being foreign-born	145/429 (34%)
Having travelled abroad during pregnancy or up to 2 months before conception	27/429 (6%)
Having a partner who has travelled abroad during pregnancy or up to 3 months before conception	23/429 (5%)
Having ever travelled to Latin America and having had high risk behavior (see Figure 1)	19/429 (4%)
Having a foreign-born mother	148/429 (34%) *
Having ever received blood transfusions abroad	0/429 (0%)

* Among the 284 Italian pregnant women 5 (2%) have a foreign-born mother (one born in Uruguay, one in the USA, one in France, one in Egypt and one in Albania). Among the 145 foreign born pregnant women 141 (97%) have a mother from the same country and 4 (3%) from a different country (in detail two Albanian pregnant women have an Italian mother, one Hungarian woman has a German mother and a Ukrainian woman has a Russian mother). Overall, there were nine cases of discordance between mother and daughter’s country of birth. In one case the pregnant lady was at risk for emerging infectious disease only because of the maternal origins. It was the case of an Italian women whose mother was born in Uruguay, which is endemic for Chagas disease and HTLV-1. The pregnant woman was consequently tested for the two infections and resulted negative.

## Data Availability

The data presented in this study are available on request from the corresponding author.

## Supplementary Materials

The following are available online at https://www.mdpi.com/2076-0817/10/1/56/s1, Table S1: List of countries at high risk for Chagas disease, HTLV-1 infection, malaria, schistosomiasis and Zika virus infection [34]. Table S2: Frequency of risk factors for five emerging infectious diseases (Chagas disease, HTLV-1 infection, malaria, schistosomiasis, Zika virus infection) in pregnant women according to their geographical area of origin.
