# Supplementary material for: Emerging Infectious Diseases in Pregnant Women in a Non-Endemic Area: Almost One Out of Four Is at Risk
Source: Pathogens. 2021 Jan 10;10(1):56. doi: 10.3390/pathogens10010056 (PMC7827164; doi:10.3390/pathogens10010056)
Supplement: Supplementary file 1 [file pathogens-10-00056-s001.pdf]

Table S1. List of countries at high risk for Chagas disease, HTLV-1 infection, malaria, schistosomiasis and Zika virus infection. [22,24,45,46]

**AFRICA**			
Angola	Malaria*, Schistosomiasis, Zika	Madagascar	Malaria* Schistosomiasis
Benin	HTLV Malaria* Schistosomiasis	Malawi	Malaria* Schistosomiasis
Botswana	Malaria*	Mali	HTLV Malaria* Schistosomiasis
Burkina Faso	HTLV Malaria* Schistosomiasis Zika	Mauritania	HTLV Malaria* Schistosomiasis
Burundi	Malaria* Schistosomiasis Zika	Mauritius	Malaria* Schistosomiasis
Cabo verde	Zika	Mozambique	HTLV Malaria* Schistosomiasis
Cameroon	HTLV Malaria* Schistosomiasis Zika	Namibia	HTLV Malaria*
Central African Republic	HTLV Malaria* Schistosomiasis Zika	Niger	Malaria* Schistosomiasis
Chad	HTLV Malaria* Schistosomiasis	Nigeria	HTLV Malaria* Schistosomiasis Zika
Comoros	Malaria* Schistosomiasis	Rwanda	Malaria* Schistosomiasis
Congo (Democratic Rep. of the Congo and Rep of the Congo)	HTLV Malaria* Schistosomiasis	Sao Tome e Principe	Malaria* Schistosomiasis
Cote d'Ivoire	HTLV Malaria* Schistosomiasis Zika	Senegal	HTLV Malaria* Schistosomiasis Zika
Djibouti	Malaria* Schistosomiasis	Liberia	HTLV Malaria* Schistosomiasis
Equatorial Guinea	HTLV Malaria* Schistosomiasis	Seychelles	HTLV Malaria* Schistosomiasis
Eritrea	Malaria*	Sierra Leone	HTLV

	Schistosomiasis		Malaria*<br>Schistosomiasis
Ethiopia	Malaria*<br>Schistosomiasis<br>Zika	Somalia	Malaria*<br>Schistosomiasis
Gabon	HTLV<br>Malaria*<br>Schistosomiasis<br>Zika	South Africa	HTLV<br>Malaria*<br>Schistosomiasis
Gambia	HTLV<br>Malaria*<br>Schistosomiasis	Sudan	Malaria*<br>Schistosomiasis
Ghana	HTLV<br>Malaria*<br>Schistosomiasis<br>Zika	Swaziland	Malaria*<br>Schistosomiasis
Guinea	HTLV<br>Malaria*<br>Schistosomiasis	Tanzania	Malaria*<br>Schistosoma
Guinea-Bissau	HTLV<br>Malaria*<br>Schistosomiasis<br>Zika	Togo	HTLV<br>Malaria*<br>Schistosomiasis
Kenya	Malaria*<br>Schistosomiasis	Uganda	Malaria*<br>Schistosomiasis<br>Zika
Lesotho	Malaria*<br>Schistosomiasis	Zambia	Malaria*<br>Schistosomiasis
		Zimbabwe	Malaria*<br>Schistosomiasis
**ASIA**			
Bangladesh	Zika	Laos	Zika
Burma (Myanmar)	Zika	Malaysia	Zika
Cambodia	Zika	Maldives	Zika
India	Zika	Philippines	Schistosomiasis<br>Zika
Indonesia	Zika	Thailand	Zika
Iran	HTLV	Vietnam	Zika
Japan	HTLV	Yemen	Schistosomiasis
**EUROPE**			
Romania	HTLV		
**LATIN AMERICA (Central America, South America and Caribbean)**			
Argentina	HTLV<br>Chagas disease<br>Zika	Haiti	HTLV<br>Zika
Belize	Chagas disease<br>Zika	Honduras	Chagas disease<br>Zika
Bolivia (Plurinational State of)	Chagas disease<br>Zika	Jamaica	HTLV<br>Zika
Brazil	HTLV<br>Chagas disease	Mexico	Chagas disease<br>Zika

	Schistosomiasis<br>Zika
Chile	HTLV<br>Chagas disease
Colombia	HTLV<br>Chagas disease<br>Zika
Costa Rica	Chagas disease<br>Zika
Cuba	Zika
Dominican Republic	HTLV<br>Zika
Ecuador	Chagas disease<br>Zika
El Salvador	Chagas disease<br>Zika
Guatemala	Chagas disease<br>Zika
Guyana and France-Guyana	HTLV<br>Chagas disease<br>Zika
Nicaragua	Chagas disease<br>Zika
Panama	HTLV<br>Chagas disease<br>Zika
Paraguay	Chagas disease<br>Zika
Perù	HTLV<br>Chagas disease<br>Zika
Suriname	HTLV<br>Chagas disease<br>Zika
Uruguay	Chagas disease
Venezuela	HTLV<br>Chagas disease<br>Zika
Other Antilles islands	Zika

*Offer malaria diagnostic test in case of: Sub-Saharan Africa native women who reached Italy ≤ 5 years ago and/or travelled to their country (or to others Sub-Saharan Africa states) in the last 5 years.

Table S2. Frequency of risk factors for five emerging infectious diseases (Chagas disease, HTLV-1 infection, malaria, schistosomiasis, Zika virus infection) in pregnant women according to their geographical area of origin.

Geographical area of origin	Women presenting at least one risk factor for one of the five selected EID (%)	Women at risk and tested for CD (%)	Women at risk and tested for HTLV-1 infection (%)	Women at risk and tested for malaria (%)	Women at risk and tested for schistosomiasis (%)	Women at risk and tested for ZIKV infection (%)
**All**	103/429 (24%)	45/429 (10%)	55/429 (13%)	11/429 (3%)	22/429 (5%)	33/429 (8%)
**Italy**	35/284 (12%)	17/284 (6%)	1/284 (< 1%)	0/284 (0%)	0/284 (0%)	20/284 (7%)
**Europe other than Italy**	21/69 (30%)	1/69 (1%)	20/69 (29%)	0/69 (0%)	0/69 (0%)	1/69 (1%)
**North America**	1/2 (50%)	0/2 (0%)	0/2 (0%)	0/2 (0%)	0/2 (0%)	1/2 (50%)
**Latin America or the Caribbean**	29/29 (100%)	27/29 (93%)	23/29 (79%)	0/29 (0%)	6/29 (21%)	6/29 (21%)
**Africa**	16/25 (64%)	0/25 (0%)	11/25 (44%)	11/25 (44%) *	16/25 (64%) #	4/25 (16%)

**Asia**	1/20 (5%)	0/20 (0%)	0/20 (0%)	0/20 (0%)	0/20 (0%)	1/20 (5%)

Footnotes. EID: Emerging infectious diseases; * one positive result; # two positive results.
